# Tuning multiple imputation by predictive mean matching and local residual draws

**DOI:** 10.1186/1471-2288-14-75

**Published:** 2014-06-05

**Authors:** Tim P Morris, Ian R White, Patrick Royston

**Affiliations:** 1 Hub for Trials Methodology Research, MRC Clinical Trials Unit at UCL, Aviation House, 125 Kingsway, WC2B 6NH, London, UK; 2 MRC Biostatistics Unit, Cambridge Institute of Public Health, Forvie Site, Robinson Way, Cambridge Biomedical Campus, CB2 0SR, Cambridge, UK

**Keywords:** Multiple imputation, Imputation model, Predictive mean matching, Local residual draws, Missing data

## Abstract

**Background:**

Multiple imputation is a commonly used method for handling incomplete covariates as it can provide valid inference when data are missing at random. This depends on being able to correctly specify the parametric model used to impute missing values, which may be difficult in many realistic settings. Imputation by *predictive mean matching* (PMM) borrows an observed value from a donor with a similar predictive mean; imputation by *local residual draws* (LRD) instead borrows the donor’s residual. Both methods relax some assumptions of parametric imputation, promising greater robustness when the imputation model is misspecified.

**Methods:**

We review development of PMM and LRD and outline the various forms available, and aim to clarify some choices about how and when they should be used. We compare performance to fully parametric imputation in simulation studies, first when the imputation model is correctly specified and then when it is misspecified.

**Results:**

In using PMM or LRD we strongly caution against using a single donor, the default value in some implementations, and instead advocate sampling from a pool of around 10 donors. We also clarify which matching metric is best. Among the current MI software there are several poor implementations.

**Conclusions:**

PMM and LRD may have a role for imputing covariates (i) which are not strongly associated with outcome, and (ii) when the imputation model is thought to be slightly but not grossly misspecified. Researchers should spend efforts on specifying the imputation model correctly, rather than expecting predictive mean matching or local residual draws to do the work.

## Background

The presence of missing data is a common issue in medical research, leading to reduced precision and sometimes bias in parameter estimates. Multiple imputation (MI) can alleviate these issues and is popular approach to dealing with missing data [1-3].

It is impossible to know for certain how data went missing. In thinking about the process there are three important scenarios [4]: 

1. *Missing completely at random* (MCAR). The probability of data being missing does not depend on observed or unobserved data.

2. *Missing at random* (MAR). Conditional on observed data, the probability of data being missing does not depend on unobserved data. MCAR is a special case of MAR.

3. *Missing not at random* (MNAR). Conditional on observed data, the probability of data being missing still depends on unobserved data.

Researchers analysing incomplete datasets should consider the process by which data may have gone missing, and perform analyses that are valid given this assumption.

MI involves specifying a parametric model for the missing data given the observed data and drawing missing values from the posterior predictive distribution *M*>1 times. This model is henceforth referred to as the *imputation model*. The *M* filled-in datasets are analysed identically according to the model that would have been used in the absence of missing data. We term this model the *analysis model*. The *M* parameter estimates are then combined using ‘Rubin’s rules’ [5].

Multiple imputation can provide valid inference given any of the above mechanisms, although standard software implementations impute assuming MAR (MCAR) by default.

If the imputation model is specified correctly, Rubin’s rules lead to consistent parameter estimation and confidence intervals that fully incorporate uncertainty due to missing data [6]. For imputing a covariate it is advisable to include in the imputation model (i) variables thought to predict missingness, (ii) variables associated with the variable being imputed, and (iii) the outcome variable of the analysis model [3,7].

One of the biggest challenges for users of MI is specifying the imputation model correctly. This is not always easy to do, even for seemingly simple analyses: for instance when the analysis model contains nonlinear functions of incomplete covariates [8].

Predictive mean matching (PMM) [9] and local residual draws (LRD) [10] are methods for drawing imputations that relax some of the assumptions of parametric imputation. In doing so they may improve robustness of inference with missing data to misspecification of the imputation model. These methods are outlined briefly below and described further in the Methods section.

For an incomplete variable *x*, an imputation model is fitted with parameters ***α*** and covariates ***z***. Parametric imputation proceeds by drawing ***α*** from its posterior distribution, before drawing missing values of *x* from the posterior predictive distribution conditional on the draw ***α***^∗^. The draws of the imputation model parameters make parametric imputation ‘proper’ [6] and may be taken parametrically or by the approximate Bayesian bootstrap [11].

PMM and LRD differ from parametric imputation as follows. Let *h* index observations with *x* observed and *j* index observations with *x* missing. For all *h*, the linear predictor ***α***^obs^ ***z***_*h*_ is calculated, and for all *j*, the linear predictor ***α***^mis^ ***z***_*j*_ is calculated (***α***^obs^ and ***α***^mis^ will be defined in the Methods section). Observed values close to the linear-predicted value are selected as the donor pool. Often, but not always, the donor pool is fixed as containing *k* candidate donors. One of these is selected at random to ‘donate’. PMM imputes the donor’s *x*_*h*_. LRD adds the donor’s residual to the recipient’s linear predictor.

In the remainder of this article, we give technical details of these methods reviewing their development and the various forms available, along with the rationale for their use. Two simulation studies on PMM and LRD are then described and reported: in the first, the imputation model is correct; in the second, the imputation model is mis-specified. We illustrate various approaches to imputing a missing covariate for a cohort study in ovarian cancer. We finish with a discussion and some conclusions.

This article describes the rationale for PMM and LRD, and their development and evaluation in previous work. They are evaluated further in some simple and then more challenging settings. Our focus is on incomplete continuous covariates, though in principle both methods may be used to impute ordinal or categorical covariates. We aim to clarify some choices about how PMM and LRD should be implemented and when they should be used.

## Methods

### The development of predictive mean matching and local residual draws

In this section, we provide a technical description of PMM and LRD, review the development of the various flavours available – of which there are several – and clarify some details. Table 1 summarises software implementations of PMM and LRD, as of February 2014, and provides some details on options for changing the default values, if available.

**Table 1:** Summary of existing software implementations of PMM and LRD

**Software**	**Method**	**Command/instructions**	**Match**	**Option to**	**Default**	**Option to specify *k*** *	**Source of information**
			**types**	**specify**	**value**		
			**available**	**match type†**	**of *k*** *		
R	PMM	mice.impute.pmm(mice package)	1	–	5	–	v2.18 documentation [12]
R	PMM	aregimpute (hmisc package)	1, 2	pmmtype = #	*n*_*h*_	kclosest = #	v3.13-0 documentation [22]
R	PMM	bbpmm (Baboon package)	?	–	?	–	v0.1-6 documentation [13]
R	PMM	mi.pmm (mi package)	?	–	?	–	v0.09-18.03 documentation [14]
SAS	PMM	regpmm (statement within proc mi)	2	–	?	K = #	SAS website [15]
SAS	PMM	midas[31]	?	–	*n*_*h*_	N/A donor selected from all *h* with probability proportional to a function of |*δ*_*hj*_|	Reference [31]
Solas	PMM	Analyze → Multiple Imputation → Predictive Mean Matching method…	0	–	10	Select ‘Use # closest cases’ option in ‘Donor pool’ tab.	Solas website [16]
SPSS	PMM	Analyze → Multiple Imputation → Impute Missing Data Values. Under the ‘Method’ tab select ‘Custom’, and under the menu for ‘Model type for scale variables’ select ‘Predictive Mean Matching (PMM)’.	?	–	1	–	SPSS website [17]
Stata	PMM	mi impute pmm	2	–	1	knn(#)	Help file for mi impute pmm[18]
Stata	PMM	ice, match	1, 2	matchtype(#)	10	matchpool(#)	Help file for ice
Stata	LRD	ice, match uvisopts(lrd)	1, 2	matchtype(#)	10	matchpool(#)	Help file for ice

^*^Type 0 matches linear predictors for observed and missing values; type 1 uses a draw of parameters for missing values before matching; type 2 uses a draw of parameters for both observed and missing.

^†^*k* is the size of the donor pool.

Both PMM and LRD begin by calculating a predictive distance *δ*_*hj*_, which can be thought of as a measure of match quality. For all *j* the *k* observations minimising |*δ*_*hj*_| are identified where

(1) $$\begin{array}{ll} \delta_{\text{hj}} & =\alpha^{\text{mis}}z_{j}-\alpha^{\text{obs}}z_{h},\; \end{array}$$

and one of these is selected at random. For PMM [9] the imputed value $x_{j}^{*}$ is taken as *x*_*h*_. For LRD [19] the imputed value $x_{j}^{*}$ is

(2) $$\begin{array}{ll} x_{j}^{*} & =\alpha^{\text{mis}}z_{j}+x_{h}-\alpha^{\text{obs}}z_{h}.\; \end{array}$$

#### Defining the matching distance

Little initially introduced PMM, suggesting the calculation of *δ*_*hj*_ such that $\alpha^{\text{mis}}=\alpha^{\text{obs}}=\hat{\alpha }$[9]. In the same article, it was noted that this did not allow for uncertainty about ***α***: in parametric imputation a draw ***α***^∗^ is taken before imputing $x_{j}^{*}$ conditional on ***α***^∗^. The use of ***α***^mis^ = ***α***^∗^ was noted as a remedy. A third metric was introduced by Heitjan and Little where ***α***^mis^ = ***α***^obs^ = ***α***^∗^[20].

We refer to these distance measures as follows:

(3) $$\begin{array}{ll} \text{Type 0 matching}\;\delta_{\text{hj}} & =\hat{\alpha }z_{j}-\hat{\alpha }z_{h}\; \end{array}$$

(4) $$\begin{array}{ll} \text{Type 1 matching}\;\delta_{\text{hj}} & =\alpha^{*}z_{j}-\hat{\alpha }z_{h}\; \end{array}$$

(5) $$\begin{array}{ll} \text{Type 2 matching}\;\delta_{\text{hj}} & =\alpha^{*}z_{j}-\alpha^{*}z_{h}\; \end{array}$$

The designation is mnemonic according to the number of * symbols appearing on the right hand side, and types 1 and 2 correspond to the designation used by the ice command in Stata [21] and the aregimpute function of the R package Hmisc[22]. Note that with a single incomplete variable *δ*_*hj*_ type 0 and type 2 are the same.

It is often difficult to determine the type of matching being used in previous work. Type 0 matching was used by David et al. [10] and Little [9], and was compared to type 2 by Schenker and Taylor [19]. Type 1 matching was described by Little [9], and White, Royston and Wood [3]. Type 2 matching has been used comparatively more (see for example [19,20,23-29]).

#### Defining the donor pool

There are three broad approaches to defining the donor pool. The first is to use a fixed number of donors *k*; the second is to define some *δ*_max_ so that any *h* for whom |*δ*_*hj*_| < *δ*_max_ are in the donor pool for *j*. This is sometimes termed ‘caliper matching’. A third approach uses *k* = *n*_*h*_, the number of observations for which *x* is observed, but is more likely to select those with small *d*_*hj*_[30,31]; see the next section.

David et al. imputed income, initially using *global* residual draws [10], setting *k* to the number of observations with *x* observed. However, the results were unsatisfactory to the authors and so *δ*_max_ = $2,000 was instead used.

The notion of selecting from a pool of potential donors was apparently not present in the work of Little [9], who matched to the nearest donor only. Heitjan and Little introduced a pool of *k* = 5 potential donors [20]; subsequent to that article authors have largely used fixed *k* > 1.

Schenker and Taylor noted the problem with defining *δ*_max_, that it is possible for a recipient to have no donors with ***α***^obs^ ***z***_*h*_ lying within ***α***^mis^ ***z***_*j*_±*δ*_max_. They suggested an adaptive method for choosing *k*, which involved defining *δ*_max_, but if *k* = 0 or 1 to set *k* = 2.

#### Sampling from the donor pool

The most common method is to randomly sample an observation from the donor pool, for example [2,19,20,24], however some more sophisticated methods have also been proposed.

Moriarity and Scheuren suggested the use of ‘constrained’ matching [32], where each *h* can only donate *x*_*h*_ once. Note that this is only feasible with less that half of values missing. An alternative, ‘slightly constrained’ matching, penalises any *h* that has already donated by reducing the probability of subsequent donation. Durrant and Skinner used a slightly constrained matching in a simulation study, and found it to be less biased than using a fixed value of *k*[33].

Siddique and Belin proposed a version of PMM that allows any *h* to donate [30], but with the probability of imputing *x*_*h*_ for individual *j* proportional to a function of |*δ*_*hj*_|. A ‘closeness’ parameter was introduced which could be altered to augment the probability of selecting the closest donors. This was later published as a SAS macro [31].

#### Notes on LRD

LRD has received far less attention than PMM. This is possibly because of the attraction that, by always borrowing observed values, PMM always imputes observable values, while LRD may not. Conversely, LRD does have the ability to impute values outside the range of observed data, and so may deal better with values that are missing in tails of a distribution.

For LRD there is a second metric to consider, unnoticed in the literature. We note the following imputation types, named correspondingly to match types:

$$\begin{array}{lll} \text{Type 0 imputation}\;x_{j}^{*} & =\hat{\alpha }z_{j}+(x_{h}-\hat{\alpha }z_{h})\; & \;\\ \text{Type 1 imputation}\;x_{j}^{*} & =\alpha^{*}z_{j}+(x_{h}-\hat{\alpha }z_{h})\; & \;\\ \text{Type 2 imputation}\;x_{j}^{*} & =\alpha^{*}z_{j}+(x_{h}-\alpha^{*}z_{h}).\; & \; \end{array}$$

With parametric imputation, $x_{j}^{*}$ are drawn from a distribution centred at ***α***^∗^*z*_*j*_. Of the above imputation metrics, only type 1 achieves this, while types 0 and 2 draw from a distribution centred at $\hat{\alpha }z_{j}$. Schenker and Taylor [19], and Barnes et al. [28] are unclear as to the imputation type used in their work.

#### Rationale for PMM and LRD

Use of PMM and LRD is typically motivated by the notion that they provide a degree of robustness when the imputation model is misspecified, for example if the normality assumption is in question, residuals are heteroscedastic, or associations are non linear.

**Figure 1:**
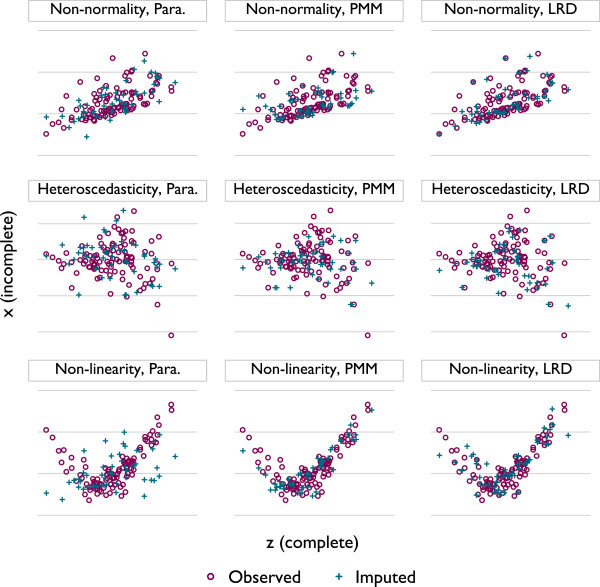
**Bivariate plots are of *x *vs. *z *values in a single imputed dataset.** Observed *x* in purple circles; imputed in blue crosses. Left to right: normal errors parametric imputation, PMM and LRD (type 1 matching with *k*=3). Top to bottom: Non-normal residuals, heteroscedasticity and non-linearity. These scenarios represent problems for a linear normal errors imputation model.

Figure 1 demonstrates how PMM and LRD may guard against these problems in 150 simulated observations, of which 50 are missing *x*, which is imputed once. The top panels show a dataset with skewed residuals, the middle panels show a dataset exhibiting heteroscedasticity, and the bottom panels show a quadratic relationship. Missing values are MCAR and imputed once by parametric draws (left panels), PMM (centre panels, type 1 matching with *k* = 3) and LRD (right panels, type 1 matching with *k*=3).

Because the data are MCAR, the missing values are a random sample of the observed values; imputed values should thus bear a close resemblance to the observed. With non-normal residuals, parametric imputation does a poor job of preserving the bivariate distribution of *y* and *x*, while PMM and LRD do a better job. In the middle panels, parametric imputation again imputes one or two values that do not match the distribution of the observed data well, while PMM borrows from the individual with the lowest observed value of *x* five times. The most stark illustration of the difference between methods is given in the lower panels, where parametric imputation seems to do a very poor job of preserving the association in observed data but PMM and LRD do well by contrast.

#### Some settings where PMM and LRD may fail

While PMM and LRD are generally advocated as methods to improve the imputation model, there are also potential weaknesses.

The price to pay for the additional flexibility supplied by PMM and LRD is that $x_{j}^{*}$ are not formally draws from the posterior predictive distribution of the imputation model; there is thus no guarantee that Rubin’s rules will be appropriate for inference.

The main specific concerns about PMM are around donor sparseness: when there are few donors with a predictive mean close to the predictive mean of a missing observation. It is clear that when |*δ*_*hj*_| is large, matches are of poor quality and so imputed values may be inappropriate. This may occur are when there are few observations with *x* observed, and under departures from MCAR.

A second pitfall for PMM arises when *δ*_*hj*_ has the same sign for all *h* in the donor pool for *j*, which will introduce a bias in the imputed values, with consequences for estimation. Again, LRD does not necessarily suffer this bias provided the direction and magnitude of residuals are appropriate.

### Simulation studies

Two simulation studies are reported below. The first compares various forms of PMM and LRD in a setting ideally suited to parametric imputation. The second compares them in a setting where parametric imputation is likely to fail. Both studies aim to evaluate type 1 versus type 2 matching, and to comment on appropriate choices of *k*.

#### Simulation design: correctly specified imputation model

In the first study, we simulate 500 observations on two variables *y* and *x* where *x*∼N(0,1) and *y*|*x* is normal in the complete data. The analysis model of interest is a linear regression

$$y_{i}∼N(\beta_{0}+\beta x_{i},100).$$

Three different strengths of *y*–*x* association are simulated: *β* = 0, *β* = 3.33 and *β* = 10, corresponding to *R*^2^ values of 0, 0.99 and 0.5 respectively.

Throughout, *y* is complete and *x* is incomplete. Three missingness mechanisms are invoked: MCAR, and two different MAR mechanisms. Let *π* denote the probability that *x* is missing. Under MCAR, *π* = 0.25. The MAR mechanisms are simulated via the linear logistic model logit(*π*) = *γ*_0_ + *γ*_1_*y*_*i*_, such that observations with large values of *y* are more likely to have values of *x* missing. Let *R* be a binary variable indicating whether *x* is not missing or missing. Values of *γ*_0_ and *γ*_1_ were chosen such that 25% of observations are missing and comparison of *R* with *y* returns an area under the ROC curve of 0.65 (‘weak’ MAR) and 0.75 (‘strong’ MAR).

The imputation model is

(6) $$\begin{array}{ll} x_{h} & ∼N(\alpha_{0}+\alpha_{1}y_{h},\sigma^{2}),\; \end{array}$$

which is correctly specified. *M*=10 imputations [6] are used for each of the following methods: 

• Parametric imputation using posterior draws.

• PMM with type 1 and type 2 matching and, for each match type, *k* = 1, 3, 5 and 10.

• LRD with type 1 and type 2 matching (type 1 imputation throughout), for each match type *k* = 1,3,5,10 and 20 (20 comes from the expectation that LRD will suffer less than PMM with larger donor pools).

The imputed datasets are analysed and estimates combined using Rubin’s rules. All imputations were produced using the ice command in Stata [21]. The various MI methods are compared to analysis of the complete data, a gold standard, and analysis of the complete cases, which any imputation method must improve upon to be worthwhile.

The whole simulation process is repeated 1,000 times. Bias, coverage of confidence intervals, and a measure of (in-)efficiency, the standard deviation of *β* over 1,000 replications (henceforth the ‘empirical standard error’), are summarised. Stata version 13 was used for all simulations [34].

#### Simulation design: misspecified imputation model

The simulation results described above evaluate PMM and LRD in a setting where we have a gold-standard imputation method. The simulation design described in this section relates to a setting where the ideal imputation method is unclear: the presence of *x* and *x*^2^ in the analysis model means it is difficult to find a compatible model for imputing *x*|*y*[35]. Here, PMM and LRD are expected to perform better than parametric imputation.

A very similar setup to the previous section is used. The key difference is that true model for the data is *x* ∼vN(1, 1) and *y* ∼ N(*βx*^2^, 10^2^). Three values of *R*^2^ used are again 0, 0.1 and 0.5. This gives a *j*-shaped relationship between *y* and *x*.

The analysis model is a normal errors linear regression,

$$y_{i}∼N(\beta_{0}+\beta_{1}x_{i}+\beta x_{i}^{2},\sigma^{2}).$$

**Figure 2:**
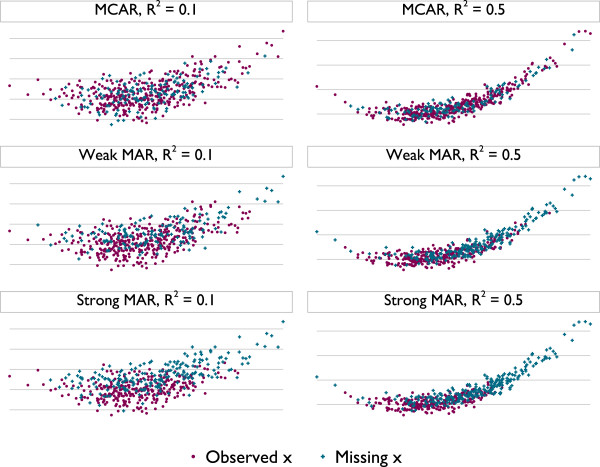
***y *** **vs. *x *** **in typical simulated datasets with a misspecified imputation model, across various simulation settings.**

The intercept and linear term are estimated even though their true values are zero. The imputation model is (6), as in the previous section. Note that no full probability model exists that accommodates both the imputation model and the analysis model [36]; this is the definition of an incompatible imputation model. Missing data are induced in the way described above. Figure 2 shows *y* and *x* in six typical simulated datasets representing the two non-zero strengths of association and three missingness mechanisms.

### Ovarian cancer example

To demonstrate PMM and LRD in practice, we provide a simple analysis of a real partially observed dataset. Clark and Altman developed a prognostic model for time to death in 1,189 individuals with epithelial ovarian cancer [37], of whom 842 died. Ten of the covariates considered for this model were incomplete, and complete cases analysis included just 518 patients. Using this dataset, we compare some of the approaches of our simulations.

One of the covariates considered by Clarke and Altman was albumin in g/dL, and was missing in 392 patients. In this dataset albumin has mean 38, standard deviation 5.3, and moderate skew of –0.52. Our analysis model is a Cox model with age in years (which is complete), albumin and albumin-squared as covariates [38].

The approaches compared are as follows: 

1. Complete cases. Analyse the subset of 797 patients with observed albumin.

2. Parametric imputation where albumin is imputed from a normal errors linear model.

3. PMM with type 2 matching and *k* = 1.

4. PMM with type 1 matching and *k* = 10.

5. LRD with type 2 matching and *k* = 1.

6. LRD with type 1 matching and *k* = 20.

The choice of settings for PMM and LRD is to reflect some of the extremes explored in our simulations. All imputation models include as covariates age, death (yes/no) and the Nelson–Aalen estimate of the cumulative hazard function [39]. For each imputation method *M* = 100 imputations were used to keep the impact of Monte Carlo error small. After imputation, albumin ^2^ was passively imputed by squaring the imputed value of albumin [3]. The Cox model was fitted in each imputed dataset and estimates combined according to Rubin’s rules [5].

## Results

### Simulation results: Correctly specified imputation model

**Figure 3:**
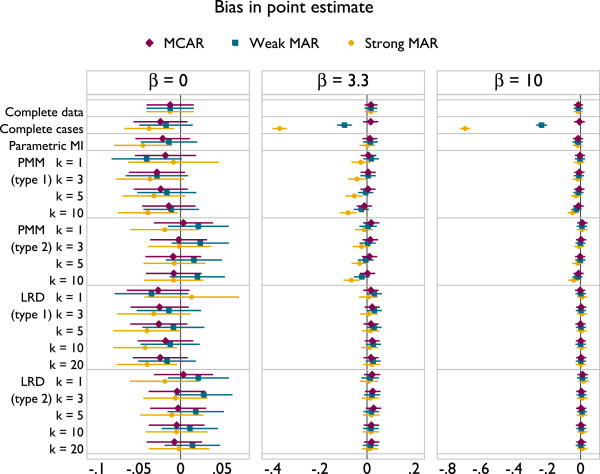
Bias under a correctly specified imputation model, according to method.

**Figure 4:**
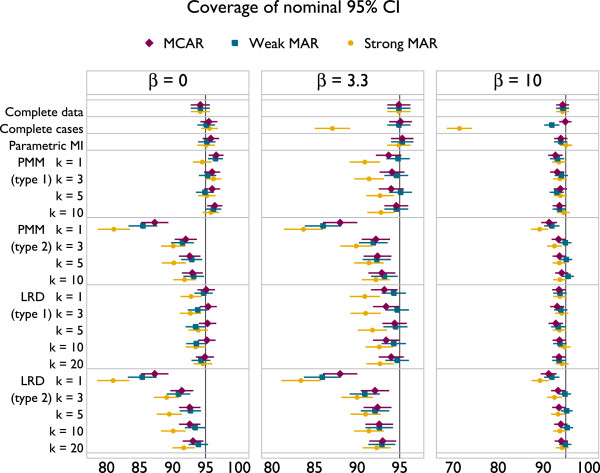
Coverage of 95% confidence intervals under a correctly specified imputation model, according to method.

**Figure 5:**
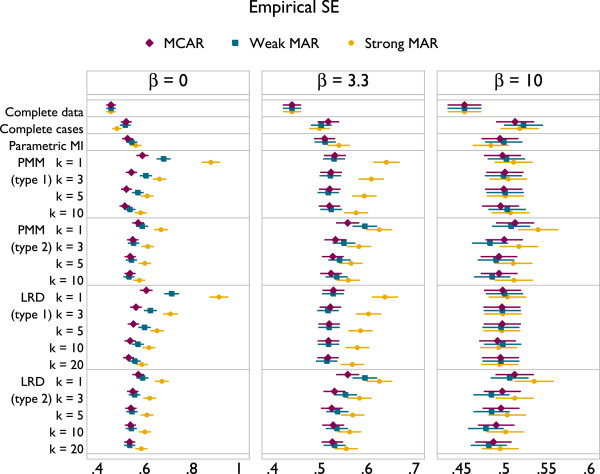
Empirical standard error of methods under a correctly specified imputation model, according to method.

Results are presented in Figures 3, 4 and 5. The plots all follow a similar design. The left panel gives results for *β* = 0, the middle panel for *β* = 3.3 and the right for *β* = 10. The different methods are labelled on the vertical axis. Results for MCAR are in purple, ‘weak’ MAR in blue and ‘strong’ MAR in orange. Point estimates are presented along with Monte Carlo 95% confidence intervals.

Results for bias are given in Figure 3. Complete cases is unbiased under MCAR and with *β* = 0, but becomes increasingly biased under the MAR mechanisms. Parametric imputation is unbiased in all scenarios as would be expected, because the imputation model is correctly specified. LRD appears to be unbiased throughout. PMM suffers a small downwards bias for *k* = 10 under strong MAR. However, the magnitude of this bias is miniscule, and it is still a vast improvement on complete cases analysis. The type of matching does not appear to have any influence on bias.

Coverage results are given in Figure 4. Again, parametric imputation performs well. PMM and LRD both tend towards under-coverage. This is worse with type 2 matching than type 1, though increasing *k* alleviates problems for both types. For type 2 matching, coverage is worse with smaller *β*.

The empirical standard errors of methods are given in Figure 5. Complete data analysis has the lowest standard errors, while complete cases and parametric imputation also tend to be low. PMM and LRD have the largest standard errors with *β* = 0 and MAR. There is a strong effect of *k* on empirical SE, with larger values of *k* never inferior to smaller values.

Taking these results together, it appears that the largest values of *k* used are optimal. There is no implication for bias with LRD, and for PMM the bias is miniscule. Coverage is always improved through larger values of *k*, as is efficiency. Type 1 matching provides better coverage than type 2 for both PMM and LRD. In scenarios where type 1 and 2 matching have comparable coverage, efficiency is also similar, although slightly lower for type 1 matching. The results for comparable forms of PMM and LRD are indistinguishable. These results can be interpreted in terms of the probability of repeated donation: if a donor is selected for many individuals within an imputation, this will lead to inefficiency; if a donor is repeatedly used by the same individuals across imputations this will lead to inefficiency and underestimation of the between-imputation variance.

### Results: Misspecified imputation model

**Figure 6:**
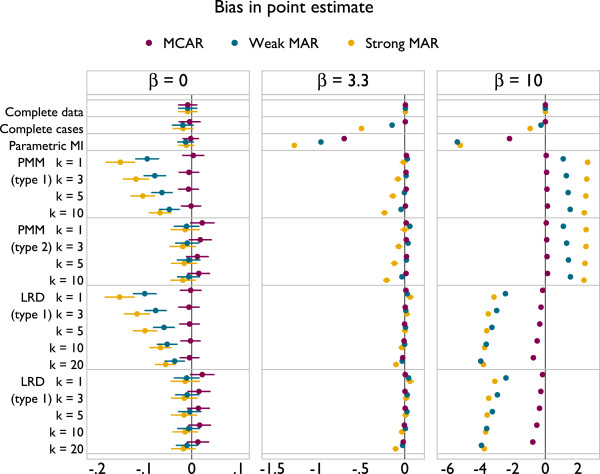
Bias under a misspecified imputation model, according to method.

**Figure 7:**
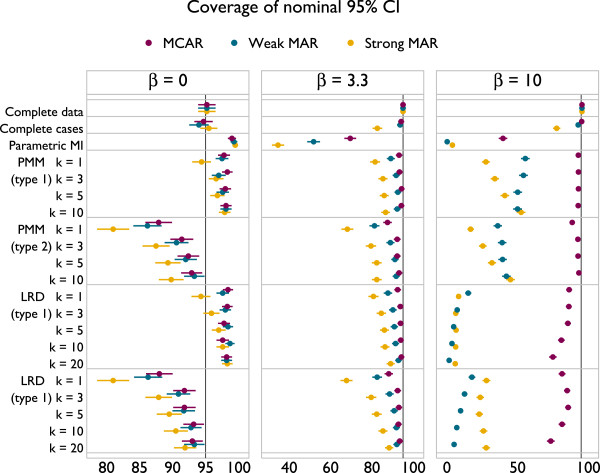
Coverage of 95% confidence intervals under a misspecified imputation model, according to method.

**Figure 8:**
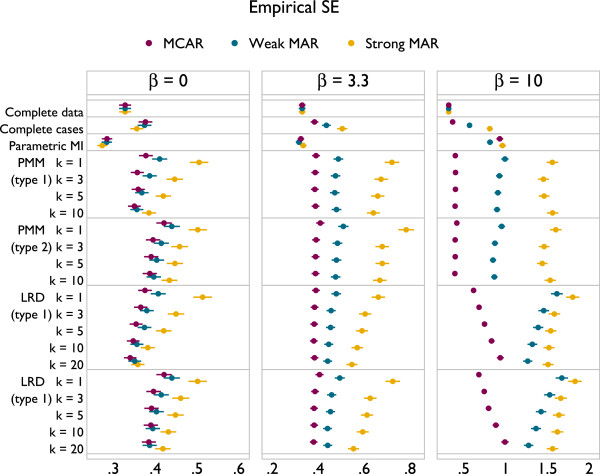
Empirical standard error of methods under a misspecified imputation model, according to method.

Results are presented in Figures 6, 7 and 8, with the design of plots following those presented in the previous section.

Parametric imputation now suffers a large bias for non-null associations, in the worst scenarios being more than half of the true value for *β*. With *β* = 0 and MAR, PMM and LRD have a very slight downwards bias for small *k* with type 1 matching. This is not present with type 2 matching. With *β* > 0 PMM and LRD always alleviate the bias seen with parametric imputation. With the ‘modest’ strength of association, *β* = 3.3, both methods have least bias with *k* = 1; as *k* increases there is a modest downwards bias under strong MAR only. In the extreme case of *β* = 10 PMM and LRD introduce a very serious degree of bias, particularly under MAR: PMM is biased away from zero and LRD towards it. To understand this bias, consider the imputed values for Figure 2. For PMM there will be a vertical spike of imputed values at the tails of the *x* distribution, while for LRD the imputed value in both tails will lie parallel to the slope of the (linear) imputation model, attenuating the degree of curvature in imputed values.

For many of the settings considered, the bias of complete cases analysis is smaller than for any of the imputation methods. For *β* = 10 this initially appears surprising, but occurs because the strong association between *y* and *x* comes close to the assumption required for complete cases analysis to be valid, that the probability of *x*_*i*_ being missing is conditionally independent of *y*_*i*_ given *x*_*i*_[40].

The coverage of imputation methods is also often poor (Figure 7). Parametric imputation gives coverage greater than 95% when *β* = 0 and much lower – close to 0% in one scenario – with *β*>0. With *β* = 0, PMM and LRD give slight over-coverage with type 1 matching, while type 2 matching gives under-coverage. For both types of matching, coverage rates increase slightly as *k* increases, as seen previously with a correctly specified imputation model. With a non-zero association between *y* and *x* and MAR, coverage can become extremely poor for all forms of PMM and LRD. For strong MAR, increasing *k* appears to slightly alleviate problems, while for weak MAR it adds to them. With *β* = 3.3 coverage for PMM and LRD are very similar, but with *β* = 10 PMM tends to give better coverage. Again, although PMM and LRD can improve upon parametric imputation the majority of the time, problems are not ‘solved’, and in the majority of settings considered complete cases analysis has better coverage.Comparison of empirical standard errors is largely unhelpful in this context because some methods have large degrees of bias. However, it is worth noting from Figure 8 that PMM and LRD are less efficient than complete cases for all settings considered here.

### Ovarian cancer example: results

Table 2 displays the log hazard ratio (HR) and 95% confidence intervals for albumin and albumin ^2^, according to method. Albumin is coded in units of 100 g/dL and centred at its mean. The log hazard ratios and confidence intervals for albumin are very similar for all methods. For albumin ^2^, the log HR is smallest for complete cases and parametric imputation, and largest for type 1 matching with large *k* (for both PMM and LRD). Note that if the inclusion of the squared term depended on its significance at the 5% level, analysis using complete cases or after parametric MI would lead to its exclusion, which is not the case for PMM and LRD.

**Table 2:** Comparison of coefficients for albumin and albumin-squared in the ovarian cancer data

	**Albumin (95% CI)**	**Albumin**^**2**^** (95% CI)**
Complete cases	–10.06 (–12.01, –8.12)	–0.18 (–0.40, 0.05)
Parametric	–10.41 (–12.45, –8.38)	–0.20 (–0.42, 0.02)
PMM, type 2, *k* = 1	–10.54 (–12.57, –8.51)	–0.25 (–0.49, –0.01)
PMM, type 1, *k* = 10	–10.74 (–12.80, –8.68)	–0.28 (–0.52, –0.04)
LRD, type 2, *k* = 1	–10.54 (–12.57, –8.51)	–0.25 (–0.49, –0.01)
LRD, type 1, *k* = 20	–10.77 (–12.75, –8.78)	–0.29 (–0.53, –0.05)

Albumin is coded in units of 100 g/dL and mean-centred.

Despite confidence intervals being of similar length for larger and smaller values of *k*, the simulation results in Figures 4 and 7 tell us that the coverage properties are rather different, and we should favour those using the larger values of *k*.

## Discussion

We have aimed to assess the performance of imputation by PMM and LRD in settings where they should perform well, and where they may perform badly. The simulation studies presented have shown that these methods can be adequate when the imputation model is correctly specified, and are an improvement over parametric imputation when the imputation model is misspecified. Nonetheless, with a misspecified imputation model, a strong association between the incomplete covariate and outcome, and data missing at random, performance can become extremely poor.

The simulation studies described and reported above involved a single incomplete covariate and a single continuous outcome. In this setting, type 2 matching is equivalent to type 0, failing to acknowledge uncertainty about the parameter of the imputation model. They demonstrate that the performance of PMM and LRD can be acceptable when the imputation model is specified correctly. When the imputation model is misspecified, they are usually an improvement over parametric imputation but can be poor nonetheless.

The design of the second simulation study was intended to provide a tough test for both methods, particularly the specific MAR mechanism used. If the mechanism had worked in the opposite direction and the sign *γ*_1_ had been negative, missing values would have occurred at lower values of *y*, which is one standard deviation from the mean of *x*.

In using PMM or LRD it is generally preferable to use type 1 matching rather than type 2 (or 0). Larger values of *k* also tend to be better in terms of coverage and efficiency. For the scenarios investigated, the largest values of *k* investigated were 10 (PMM) and 20 (LRD). However in much larger datasets with tens of thousands of observed data points, much larger values of *k* might be considered.

PMM has a cosmetic advantage over LRD that it always imputes observable values meaning it is attractive for imputing non-continuous variables. Table 1 shows that at the time of writing, this is impossible in the majority of software implementations. Only aregimpute in R and ice in Stata have type 1 matching and allow the user to specify *k*. Further, ice is the only existing software implementation of LRD.

The main problems with PMM are related to donor sparsity – with few donors in the vicinity of an incomplete case, the imputed values may lead to bias. This also applies to LRD when the imputation model is misspecified. Donor sparsity is expected when there is a large proportion of missing data, under MAR, and in the tails of distributions. PMM also suffers from bias when *δ*_*hj*_ has the same sign for all donors in the pool.

In general, the recent work by Bartlett et al. [35] may be more fruitful for multiple imputation of incomplete covariates where the analysis model contains nonlinear functions of these. We also note the recent method of Vink and van Buuren as an alternative approach to imputing squares [41].

## Conclusions

We conclude that PMM and LRD may have a role for imputing covariates when the imputation model is thought to be slightly misspecified, but researchers should focus attention on specifying the imputation model correctly, for example using the recent method described in [35], rather than expecting PMM or LRD to do the hard work.

## Competing interests

The authors declare that they have no competing interests.

## Authors’ contributions

This research was conceived by IRW and PR. All authors contributed to the design and interpretation of simulation studies. TPM performed the simulations and the illustrative analysis, and drafted the manuscript. All authors have approved the submitted version.

## Pre-publication history

The pre-publication history for this paper can be accessed here:

http://www.biomedcentral.com/1471-2288/14/75/prepub

## References

[B1] Harel O Zhou XH **Multiple imputation: review of theory, implementation and software** Stat Med 2007 26 3057 3077 10.1002/sim.2787 17256804

[B2] Horton NJ Kleinman KP **Much ado about nothing: A comparison of missing data methods and software to fit incomplete data regression models** Am Stat 2007 61 79 90 10.1198/000313007X172556 17401454 PMC1839993

[B3] White IR Royston P Wood AM **Multiple imputation using chained equations: Issues and guidance for practice** Stat Med 2011 30 4 377 399 10.1002/sim.4067 21225900

[B4] Rubin DB **Inference and missing data** Biometrika 1976 63 581 592 10.1093/biomet/63.3.581

[B5] Rubin DB Multiple Imputation for Nonresponse in Surveys 1987 New York: John Wiley and Sons

[B6] Schafer JL **Multiple imputation: a primer** Stat Methods Med Res 1999 8 1 3 15 10.1191/096228099671525676 10347857

[B7] Moons K Donders R Stijnen T Harrel F **Using the outcome for imputation of missing predictor values was preferred** J Clin Epidemiol 2006 59 10 1092 1101 10.1016/j.jclinepi.2006.01.009 16980150

[B8] Seaman SR Bartlett JW White IR **Multiple imputation of missing covariates with non-linear effects and interactions: an evaluation of statistical methods** BMC Med Res Methodol 2012 12 1 46+ 10.1186/1471-2288-12-46 22489953 PMC3403931

[B9] Little RJA **Missing-data adjustments in large surveys** J Business & Econ Stat 1988 6 287 296

[B10] David M Little RJA Samuhel ME Triest RK **Alternative methods for CPS income imputation** J Am Stat Assoc 1986 81 393 29 41 10.1080/01621459.1986.10478235

[B11] Rubin DB Schenker N **Multiple imputation for interval estimation from simple random samples with ignorable nonresponse** J Am Stat Assoc 1986 81 366 374 10.1080/01621459.1986.10478280

[B12] van Buuren S Groothuis-Oudshoorn K Mice: Multivariate Imputation by Chained Equations February 2014 Netherlands Organisation for Applied Scientific Research TNO

[B13] Meinfelder F BaBooN: Bayesian Bootstrap Predictive Mean Matching – Multiple and single imputation for discrete data March 2011 Universität Bamberg

[B14] Gelman A Hill J Su YS Yajima M Pittau MGmi: Missing Data Imputation and Model Checking August 2013 Columbia University

[B15] SAS Institute Inc **Predictive mean matching method for monotone missing data** February 2014 http://support.sas.com/documentation/cdl/en/statug/63033/HTML/default/viewer.htm#statug_mi_sect020.htm

[B16] Solas for Missing Data Analysis **Predictive mean matching method** February 2014 http://www.statsols.com/predictive-mean-matching-method/

[B17] SPSS **Predictive mean matching (multiple imputation algorithms)** February 2014 http://pic.dhe.ibm.com/infocenter/spssstat/v20r0m0/index.jsp?topic=%2Fcom.ibm.spss.%20statistics.help%2Falg_ multiple_imputation_univariate_pmm.htm

[B18] StataCorp **mi impute pmm** February 2014 http://www.stata.com/manuals13/mimiimputepmm.pdf

[B19] Schenker N Taylor JMG **Partially parametric techniques for multiple imputation** Comput Stat & Data Anal 1996 22 4 425 446 10.1016/0167-9473(95)00057-7

[B20] Heitjan DF Little RJA **Multiple imputation for the fatal accident reporting system** J R Stat Soc Series C (Appl Stat) 1991 40 1 13 29

[B21] Royston P **Multiple imputation of missing values: update** Stata J 2005 5 527 536

[B22] Harrell FE Hmisc: Harrell Miscellaneous January 2014 Vanderbilt University

[B23] Heitjan DF Landis RJ **Assessing secular trends in blood pressure: a multiple-imputation approach** J Am Stat Assoc 1994 89 427 750 759 10.1080/01621459.1994.10476808

[B24] Zhou XH Eckert GJ Tierney WM **Multiple imputation in public health research** Stat Med 2001 20 9-10 1541 1549 10.1002/sim.689 11343373

[B25] Horton NJ Lipsitz SR **Multiple imputation in practice: comparison of software packages for regression models with missing variables** Am Stat 2001 55 244 254 10.1198/000313001317098266

[B26] Tang L Song J Belin TR Unützer J **A comparison of imputation methods in a longitudinal randomized clinical trial** Stat Med 2005 24 14 2111 2128 10.1002/sim.2099 15889392

[B27] Hsu CH Taylor JMG Murray S Commenges D **Survival analysis using auxiliary variables via non-parametric multiple imputation** Stat Med 2006 25 20 3503 3517 10.1002/sim.2452 16345047

[B28] Barnes SA Lindborg SR Seaman JW **Multiple imputation techniques in small sample clinical trials** Stat Med 2006 25 2 233 245 10.1002/sim.2231 16220515

[B29] Qi L Wang Y-FF He Y **A comparison of multiple imputation and fully augmented weighted estimators for Cox regression with missing covariates** Stat Med 2010 29 25 2592 2604 10.1002/sim.4016 20806403 PMC4022355

[B30] Siddique J Belin TR **Multiple imputation using an iterative hot-deck with distance-based donor selection** Stat Med 2008 27 1 83 102 10.1002/sim.3001 17634973

[B31] Siddique J Harel O **MIDAS: a SAS macro for multiple imputation using distance-aided selection of donors** J Stat Softw 2009 29 9 1 18

[B32] Moriarity C Scheuren F **A note on rubin’s statistical matching using file concatenation with adjusted weights and multiple imputations** J Business & Econ Stat 2003 21 1 65 73 10.1198/073500102288618766

[B33] Durrant GB Skinner C **Using missing data methods to correct for measurement error in a distribution function** Surv Methodol 2006 32 1 25 36

[B34] StataCorp Stata Statistical Software: Release 13 2013 College Station, TX: Stata Press

[B35] Bartlett JW Seaman SR White IR Carpenter JR for the Alzheimer’sDiseaseNeuroimagingInitiative* **Multiple imputation of covariates by fully conditional specification: accommodating the substantive model** Stat Methods Med Res 2014 0962280214521348+ http://smm.sagepub.com/content/early/2014/03/31/0962280214521348 10.1177/0962280214521348 PMC4513015 24525487

[B36] Morris TP White IR Royston P Seaman SR Wood AM **Multiple imputation for an incomplete covariate that is a ratio** Stat Med 2014 33 1 88 104 10.1002/sim.5935 23922236 PMC3920636

[B37] Clark TG Altman DG **Developing a prognostic model in the presence of missing data** J Clin Epidemiol 2003 56 1 28 37 10.1016/S0895-4356(02)00539-5 12589867

[B38] Cox DR **Regression models and life tables** J R Stat Soc series B 1972 34 187 220

[B39] White IR Royston P **Imputing missing covariate values for the cox model** Stat Med 2009 28 15 1982 1998 10.1002/sim.3618 19452569 PMC2998703

[B40] Dardanoni V Modica S Peracchi F **Regression with imputed covariates: A generalized missing-indicator approach** J Econom 2011 162 2 362 368 10.1016/j.jeconom.2011.02.005

[B41] Vink G van Buuren S **Multiple imputation of squared terms** Sociol Methods & Res 2013 42 4 598 607 10.1177/0049124113502943

